# Enhanced propagation of *Granulicatella adiacens* from human oral microbiota by hyaluronan

**DOI:** 10.1038/s41598-022-14857-9

**Published:** 2022-06-29

**Authors:** Shun Yabuuchi, Sayoko Oiki, Shuma Minami, Ryuichi Takase, Daisuke Watanabe, Wataru Hashimoto

**Affiliations:** Laboratory of Basic and Applied Molecular Biotechnology, Division of Food Science and Biotechnology, Graduate School of Agriculture, Kyoto University, Uji, Kyoto 611-0011 Japan

**Keywords:** Microbiology, Carbohydrates, Enzymes

## Abstract

Host determinants for formation/composition of human oral microbiota remain to be clarified, although microorganisms entering the mouth cannot necessarily colonize the oral environment. Here we show that human oral-abundant bacteria degraded host glycosaminoglycans (GAGs) in saliva and gingiva, and certain bacteria significantly grew on hyaluronan (HA), a kind of GAGs. Microbial communities from teeth or gingiva of healthy donors assimilated HA. Metagenomic analysis of human oral microbiota under different carbon sources revealed HA-driven *Granulicatella* growth. HA-degrading bacterial strains independently isolated from teeth and gingiva were identified as *Granulicatella adiacens* producing extracellular 130 kDa polysaccharide lyase as a HA-degrading enzyme encoded in a peculiar GAG genetic cluster containing genes for isomerase KduI and dehydrogenase DhuD. These findings demonstrated that GAGs are one of the host determinants for formation/composition of oral microbiota not only for colonization but also for the adaptation to the host niche. Especially, HA enhanced the *G. adiacens* propagation.

## Introduction

Human oral microbiota is composed of hundreds of bacterial species working as an indigenous natural defense system^1,2^. Although a variety of microorganisms can enter the mouth through food, air, water, and human/animal contacts, the oral microbiota composition is stably maintained^3^. Disturbances in the balance of the oral ecosystem have impact on tooth decay, periodontitis, oral mucosal diseases, and severe systemic effects, including cardiovascular and neurological diseases^4,5^. The formation/composition and maintenance of the oral microbiota should be precisely understood to prevent and treat oral infections.

Periodontitis is a soft tissue-inflammation, an oral, but not a dental (tooth-, hard-tissue-associated) disease, and not defined as a bacterial infection anymore^6^. However, “pathobionts”, previously called as periodontitis-associated bacteria, partially play different and synergistic roles in dysbiosis among oral microbiota^7^. Some of the “pathobionts” need potent proteases that degrade immunoglobulins, complement, and other proteinous defense lines to invade the epithelium para-/intra-cell^8^. Besides, some oral bacteria produce enzymes degrading the extracellular matrix (ECM), the noncellular scaffolding framework important for development, tissue homeostasis, and injury responses^9–11^. ECM is composed of an interlocking mesh of fibrous proteins and glycosaminoglycans (GAGs)^12,13^, linear sulfated polysaccharides [e.g., chondroitin sulfate C (CSC), heparin (HP), and heparan sulfate] consisting of repeating disaccharide units of a uronic sugar and an amino sugar. Hyaluronan (hyaluronic acid, HA), composed of glucuronate and *N*-acetyl glucosamine (GlcNAc), a highly abundant component in ECM, is a unique nonsulfated GAG, not covalently attached to the proteins^11,14,15^. Several GAGs are frequently detected in the oral environment, such as saliva^14^ and gingiva^16^.

HA-degrading lyases, which depolymerize HA to unsaturated disaccharides, cause rapid and severe infections and thus are called spreading factors^17,18^. For example, streptococci distributed in various tissues, such as the oral, gut, and urogenital organs, are known to degrade and/or assimilate HA^19,20^, and streptococcal HA lyases release unsaturated HA disaccharides from HA^21^. Previous reports have indicated that unsaturated HA disaccharides are incorporated into bacterial cells by phosphotransferase system (PTS)^22^ and degraded and metabolized to glyceraldehyde-3-phosphate and pyruvate by cytoplasmic enzymes^23–25^, such as unsaturated glucuronyl hydrolase (UGL), 4-deoxy-l-*threo*-5-hexosulose-uronate ketol-isomerase (DhuI), 2-keto-3-deoxy-d-gluconate dehydrogenase (DhuD), 2-keto-3-deoxy-d-gluconate kinase (KdgK), and 2-keto-3-deoxy-d-phosphogluconate aldolase (KdgA; Supplementary Fig. 1a). These proteins or enzymes are encoded in a GAG genetic cluster^23^ often conserved in the human microbiota (Supplementary Fig. 1b), suggesting that GAG utilization is involved in formation/composition of microbiota on tissues containing GAGs^26^.

Previous studies have revealed that gut microbiota, particularly gut-abundant *Bacteroides* species, degrades and assimilates GAGs as nutrient sources^26–28^. Although exogenously supplied nutrients from food are highly variable in both quality and quantity, host-derived GAGs, which are independent on intake of meals, are constitutively produced as a component of ECM in human intestine. Thus, utilization of GAGs is considered a survival and adaptation strategy for *Bacteroides* species^29^. In contrast, degradation of GAGs by oral bacteria has been studied mainly from the viewpoint of host infection. Little knowledge on oral bacteria utilizing GAGs for survival and adaptation in the oral environment has been accumulated. This article deals with the significance of GAG utilization as a host adaptation strategy by the human oral microbiota ecosystem through metagenomics and GAG degradation/assimilation assays. Especially, *Granulicatella adiacens* remarkably grew on HA and equipped the peculiar gene cluster for GAG degradation, import, or metabolism in the bacterial genome through the molecular identification of HA lyase.

## Results and discussion

### GAG degradation by oral-abundant bacteria

**Figure 1:**
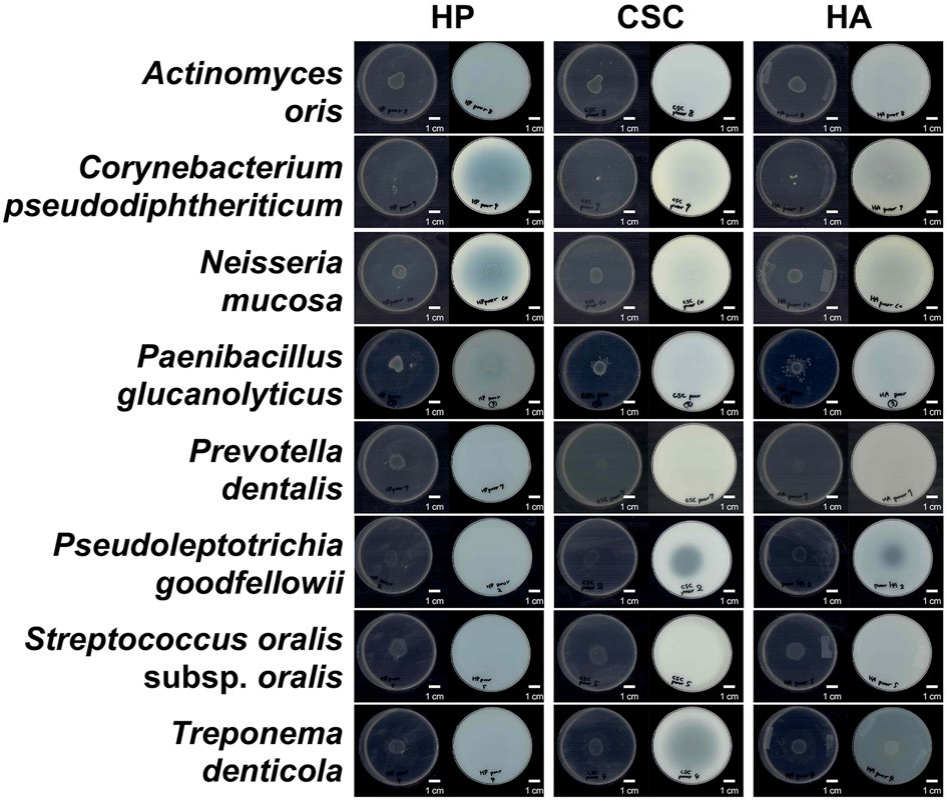
Glycosaminoglycan (GAG) degradation by typical oral bacteria. Eight typical oral bacterial species from different genera were grown on the center of the halo-forming minimal medium plate containing heparin (HP) (left), chondroitin sulfate C (CSC) (middle), or hyaluronan (HA) (right). After full growth on the medium plate (left), acetic acid was spread onto the plate for halo formation (right). Bar, 1 cm. Only *N. mucosa* degraded HP. *P. goodfellowii* and *T. denticola* degraded CSC and HA.

Eight typical species frequently detected in human oral microbiota^30–33^ (*Actinomyces oris*, *Corynebacterium pseudodiphtheriticum*, *Neisseria mucosa*, *Paenibacillus glucanolyticus*, *Prevotella dentalis*, *Pseudoleptotrichia goodfellowii*, *Streptococcus oralis* subsp. *oralis*, and *Treponema denticola*) were subjected to a halo assay for GAG degradation^34^. Bacterial cells spotted on the center of the agar plate containing GAG showed a clear zone, indicating GAG degradation. On nutrient-poor agar plates (Fig. 1), HP degradation was observed only in *Neisseria mucosa*. CSC and HA were clearly degraded by *P. goodfellowii* and *T. denticola*. On nutrient-rich agar plates (Supplementary Fig. 2), no HP degradation was detected in the tested bacteria. CSC was degraded by *P. goodfellowii* and *T. denticola*, and HA was degraded by *T. denticola*. Combined with the gene array data that genes coding for GAG-degrading enzymes such as *N*-acetyl-d-galactosamine-4-sulfate 4-sulfohydrolase, β-*N*-acetyl-d-hexosaminide *N*-acetylhexosaminohydrolase, and *N*-acetyl-d-glucosamine-6-sulfate 6-sulfohydrolase are enriched in oral microbiota of patients with periodontitis^30^, these results suggested that several oral-abundant bacterial genera include GAG-degrading species.

**Figure 2:**
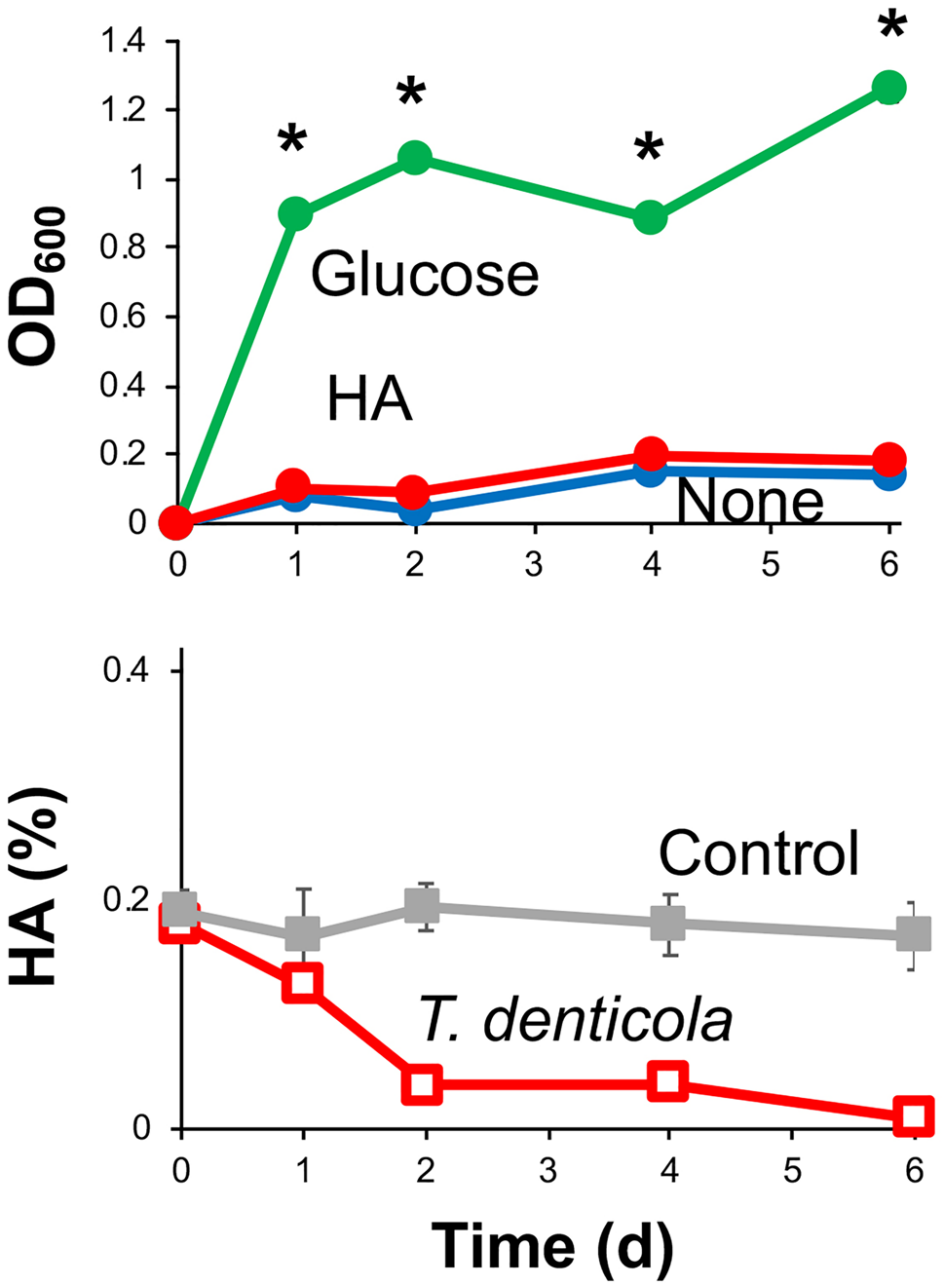
HA assimilation by *T. denticola*. Growth of *T. denticola* in nutrient-poor medium (1/20 × Gifu anaerobic medium (GAM); blue) or in the same liquid medium but containing glucose (green) or HA (red) as a carbon source (top). *p < 0.05, significant growth promotion compared to growth in nutrient-poor medium (*t* test). HA concentrations during growth in HA-containing medium were determined (bottom). *T. denticola* degraded HA.

Growth in liquid medium containing glucose or HA as a carbon source was examined with *T. denticola* (Fig. 2). Although *T. denticola* showed only weak growth without addition of saccharides, the bacterial cells vigorously proliferated and reached the stationary phase within 1 day after inoculation when glucose was added as a carbon source. Intriguingly, *T. denticola* completely degraded HA within 2 days without showing assimilation. In fact, oral spirochaetes, including *T. denticola*, were reported to degrade HA and CSC by an extracellular enzyme^35^. The results suggested that some oral-abundant bacteria (e.g., *T. denticola*) degrade GAGs specifically for invasive penetration into host tissues.

### HA degradation and assimilation by oral microbiota

**Figure 3:**
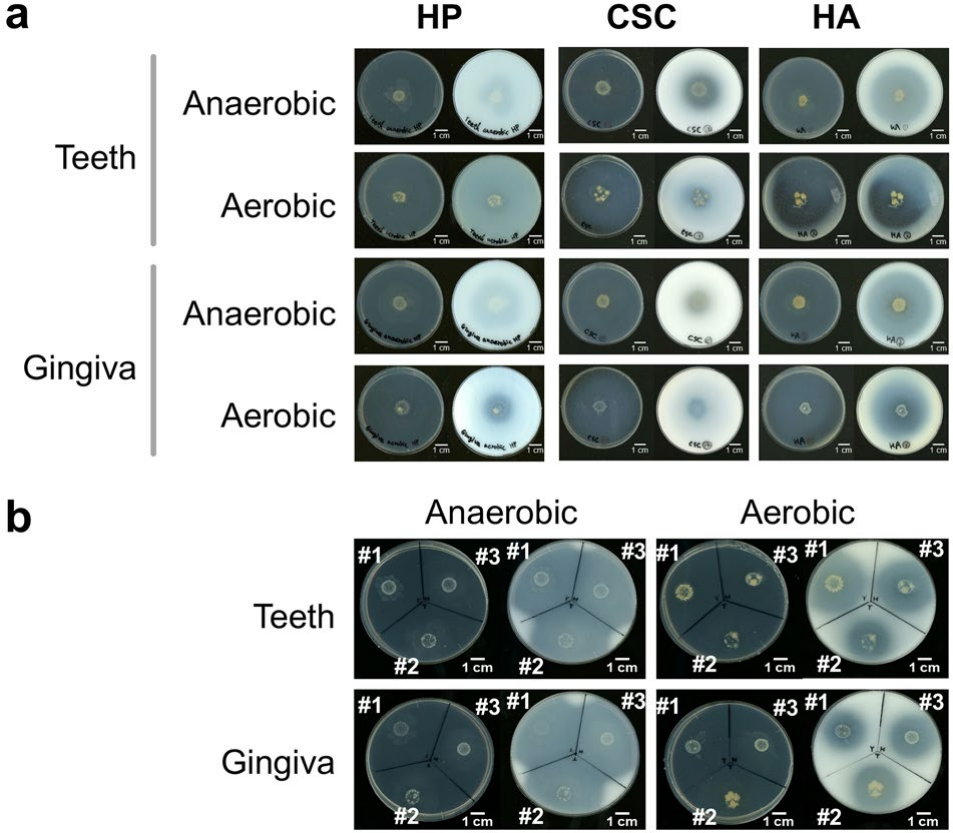
GAG degradation by human oral microbiota. (**a**) Microbial community from teeth or gingiva of donor #1 was grown anaerobically or aerobically on the center of the halo-forming minimal medium plate containing HP (left), CSC (middle), or HA (right). (**b**) Microbial communities from teeth or gingiva of donors #1 to #3 were grown anaerobically or aerobically on the center of the halo-forming minimal medium plate containing HA. After full growth on the medium plate (left), acetic acid was spread onto the plate for halo formation (right). Bar, 1 cm. The oral microbiota from teeth or gingiva of healthy donors degraded HA under both anaerobic and aerobic conditions.

Based on GAG degradation by typical oral bacteria, a part of oral microbiota may utilize host GAGs for adaptation. Thus, GAG degradation and assimilation by oral microbiota were examined using human samples. The microbial community from teeth or gingiva of a healthy donor (hereafter referred to as donor #1) showed significant HA-degrading activity under both anaerobic and aerobic conditions (Fig. 3a). The microbiota degraded CSC to a lesser extent. Weak HP degradation was only observed in the gingiva sample under aerobic condition. Thin-layer chromatography (TLC) analysis also indicated that the oral microbial community of donor #1 degraded HA, CSC, and HP at a similar level to the halo assay (Supplementary Fig. 3). HA degradation was reproducibly observed using different samples from healthy donors #2 and #3 (Fig. 3b). Thus, every oral sample from teeth and gingiva of three donors degraded HA, indicating that HA-degrading bacteria are involved in formation/composition of oral microbiota.

**Figure 4:**
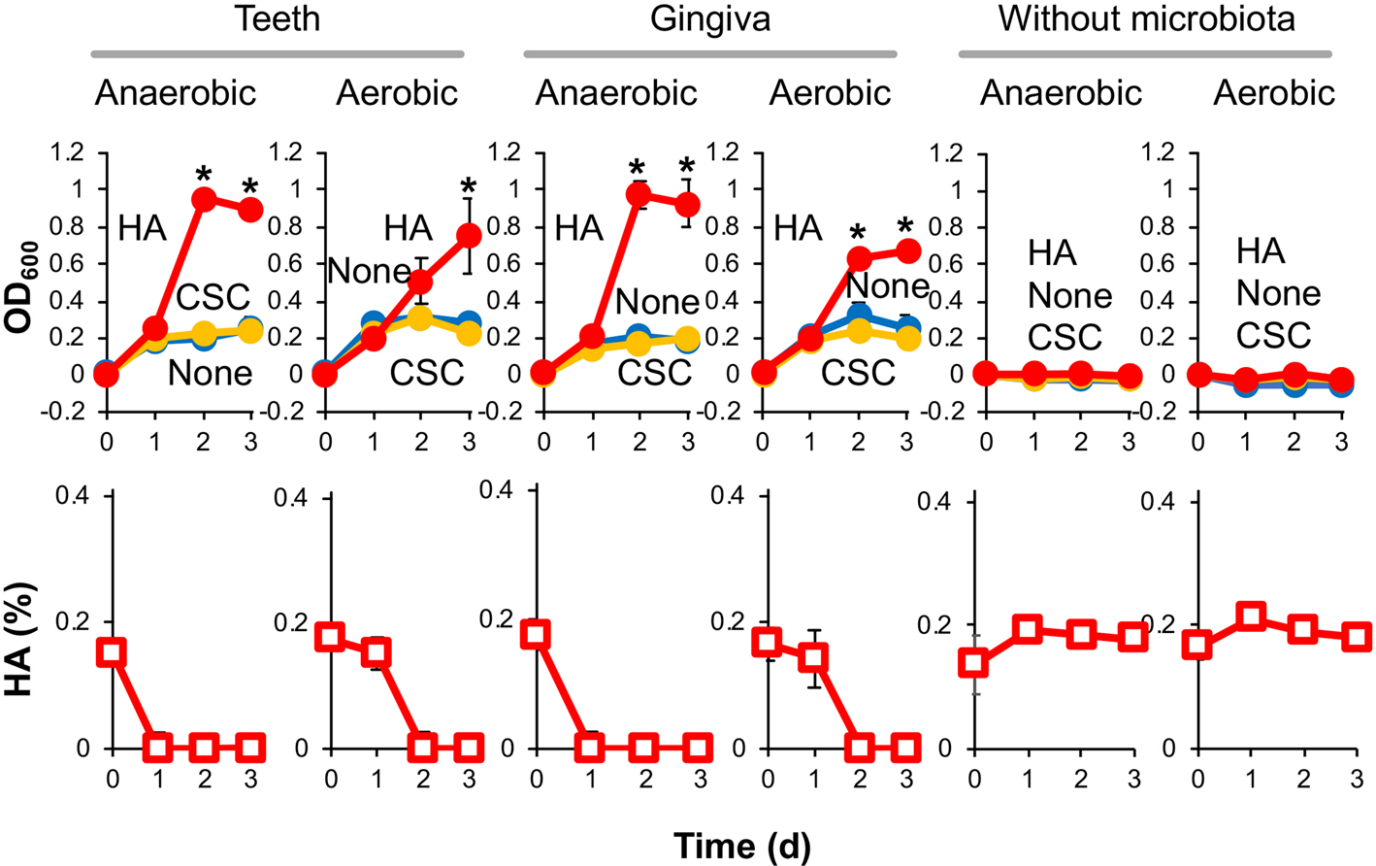
HA and CSC assimilation by human oral microbiota. Growth of microbial community from the teeth (left) or gingiva (center) of donor #1 in nutrient-poor medium (1/20 × GAM; blue) or the same liquid medium but containing HA (red) or CSC (yellow) as a carbon source (top). *p < 0.05, significant growth promotion compared to growth in nutrient-poor medium (1/20 × GAM; *t* test). HA concentrations during growth in HA-containing medium were determined (bottom). These experiments were also carried out without human oral microbiota as a negative control (right). The oral microbiota from teeth or gingiva of a healthy donor assimilated HA under both anaerobic and aerobic conditions.

Bacterial growth in CSC or HA-containing liquid medium was examined after inoculation of the teeth or gingiva sample of donor #1 (Fig. 4). Although no significant growth was observed in CSC medium, HA degradation promoted the growth of oral microbiota from both teeth and gingiva under anaerobic and aerobic conditions. Compared to HA, CSC was less effectively and reproducibly degraded by oral microbiota because full degradation of CSC in the medium needed a long time, resulting in no promotion of the bacterial growth. This typical result is shown in Supplementary Fig. 4. Incubation without microbiota as a negative control showed no degradation of HA or CSC under anaerobic and aerobic conditions. These data raised the possibility that HA is a specific target for assimilation and degradation by human oral microbiota.

### HA-adapted growth of oral microbiota

**Figure 5:**
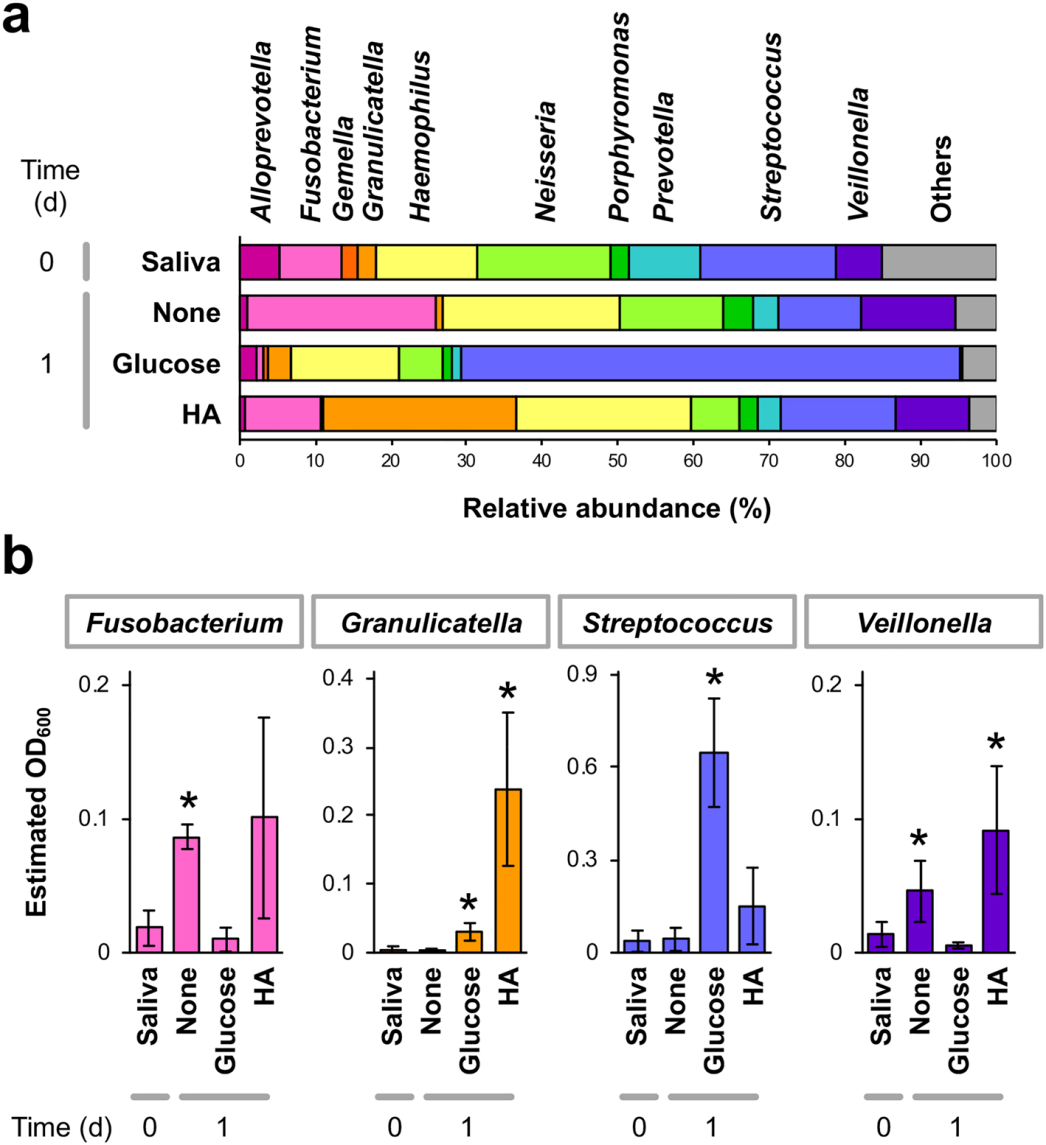
Metagenomic analysis of human oral microbiota cultivated under different carbon sources. Saliva samples from donors #1 to #3 were anaerobically grown in nutrient-poor medium (1/20 × GAM) or the same liquid medium but containing glucose or HA as a carbon source. (**a**) Genera frequency profiles averaged among three donors. Saliva samples before inoculation and the microbial communities after 1-day cultivation were subjected to 16S rRNA gene-based metagenomic analysis. (**b**) Estimated growth profiles of *Fusobacterium*, *Granulicatella*, *Streptococcus*, and *Veillonella* genera in the microbial communities, averaged among three donors. Growth was estimated by multiplying the occupancy and whole optical density at 600 nm (OD_600_) value. *p < 0.05, significant growth promotion compared to the original saliva sample (*t* test). HA promoted the growth of *Granulicatella*.

To identify the members of oral microbiota utilizing host HA for adaptation to the oral environment, metagenomics analysis was performed using three human samples. Saliva samples of healthy donors #1 to #3 were inoculated into nutrient-poor liquid medium with or without the addition of glucose or HA and cultivated under anaerobic conditions. The sample of donor #1 was also grown aerobically. Whole bacterial growth was promoted by both glucose and HA, reaching the stationary phase within 1 day after inoculation (Supplementary Fig. 5). Saliva samples before inoculation and microbial communities after 1-day cultivation were subjected to 16S rRNA gene-based metagenomic analysis. The average data of donors #1 to #3 under anaerobic conditions exhibited nutrient-dependent alterations in the oral microbiota composition (Fig. 5a). In the original saliva sample, *Streptococcus* (18.1% of occupancy), *Neisseria* (17.4%), *Haemophilus* (13.6%), *Prevotella* (9.3%), *Fusobacterium* (8.2%), and *Veillonella* (6.2%) were major predominant genera. After 1-day cultivation in nutrient-poor medium, *Fusobacterium* (24.8%), *Haemophilus* (23.5%), and *Veillonella* (12.3%) increased the occupancy. Glucose predominantly promoted the growth of *Streptococcus* species (65.8%), whereas *Granulicatella* (25.5%), *Haemophilus* (23.0%), *Fusobacterium* (10.2%), and *Veillonella* (9.9%) showed higher abundance in HA-containing medium than in the original saliva samples.

The growth of bacterial species belonging to each genus was estimated by multiplying the average occupancy and the whole-cell density (optical density at 600 nm, OD_600_; Fig. 5b). As a result, an obvious increase in the estimated OD_600_ value after 1-day cultivation in HA-containing medium was detected in *Fusobacterium* (4.1-fold), *Granulicatella* (51.2-fold), *Streptococcus* (6.7-fold), and *Veillonella* (5.5-fold). Among these genera, the growth of *Fusobacterium* and *Veillonella* was also promoted without the addition of glucose or HA, suggesting that they adapt to a certain component in nutrient-poor medium. *Streptococcus* showed a higher estimated OD_600_ after cultivation in glucose-containing medium (17.5-fold) than in HA-containing medium. In contrast, the growth of *Granulicatella* was particularly promoted by the addition of HA. The HA-dependent growth promotion of *Granulicatella* was commonly observed in all donors’ saliva samples (Supplementary Fig. 6). HA treatment has been reported to decrease *Granulicatella* in peri-implantitis^36^, although such a decrease occurs only in a specific oral microbiota, but not in the others. Our results obtained from all of the oral samples from healthy donors demonstrated that the oral microbiota composition is partly determined by available nutrients in the oral environment and that host HA is utilized as a constant energy source by specific oral bacteria, such as *Granulicatella* species, for survival and adaptation in the human oral niche.

### HA-assimilating bacteria isolated from oral microbiota

**Figure 6:**
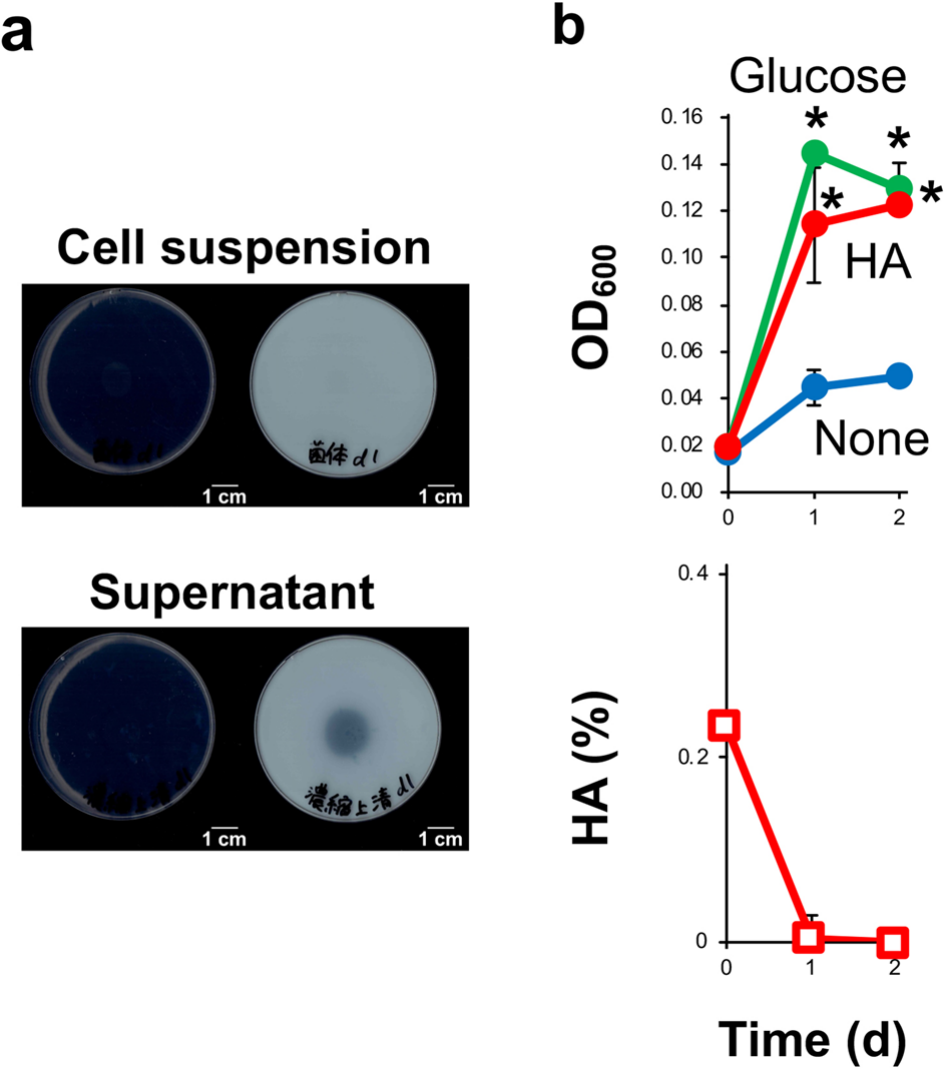
Isolation of HA-utilizing bacteria from human oral microbiota. (**a**) HA degradation by *G. adiacens*. Cell suspension or supernatant was spotted on the center of the halo-forming plate for 1 day (left). Acetic acid was spread onto the plate for halo formation (right). (**b**) Assimilation assay of HA by *G. adiacens*. Growth of *G. adiacens* in nutrient-poor medium (1/20 × GAM; blue) or the same liquid medium but containing glucose (green) or HA (red) as a carbon source was compared (top). *p < 0.05, significant growth promotion compared to growth in nutrient-poor medium (1/20 × GAM; *t* test). HA concentrations during growth in HA-containing medium were determined (bottom). The isolated *G. adiacens* extracellularly secreted HA-degrading enzymes and assimilated HA.

Oral microbiota from teeth or gingiva of uninfected donor #1 was cultivated on HA-containing agar plates under anaerobic or aerobic conditions. Based on the halo assay, one clone derived from teeth and two clones derived from gingiva cultivated under anaerobic conditions were isolated as HA-degrading oral bacteria. Based on the nucleotide sequence of the 16S rRNA gene, all three isolated HA-degrading bacteria were identified to be a member of *G. adiacens*, Gram-positive commensal bacteria in the human oral cavity and gastrointestinal tract. The HA-degrading activity of the isolated *G. adiacens* culture was derived from the supernatant but not detected in saline-washed cells (Fig. 6a), indicating that *G. adiacens* secretes HA-degrading enzymes to the extracellular environment directly and/or via membrane vesicles. In the nutrient-poor liquid medium containing HA as a major carbon source, HA degradation coincided with the growth of *G. adiacens* (Fig. 6b). The growth level of *G. adiacens* in the nutrient-rich liquid medium containing HA was comparable to that in the nutrient-rich liquid medium without HA (Supplementary Fig. 7a). On the other hand, in the presence of *G. adiacens*, intensity of the spot corresponding to unsaturated HA disaccharide was found to increase in time-dependent manner by TLC analysis (Supplementary Fig. 7b), indicating that HA was periodically degraded to unsaturated HA disaccharide. This result indicates the possibility that *G. adiacens* can degrade HA in oral cavity, where nutrients are constantly brought by food, saliva, tissue exudates, crevicular fluids, or degenerating host cells. Together with metagenomic data (Fig. 5), these results demonstrated that *G. adiacens* secretes HA-degrading enzymes to assimilate HA as a nutrient source. As a noncausal bacterium for oral diseases, *G. adiacens* degraded and assimilated HA to adapt to the oral environment.

### Molecular identification of HA lyase in *G. adiacens*

Because HA-degrading enzymes were unidentified in *G. adiacens*, the bacterial enzyme with HA lyase activity was purified to homogeneity from the 2 L culture supernatant of *G. adiacens* through ammonium sulfate fractionation and two kinds (anion exchange and gel filtration) of column chromatography (Table 1). On the sodium dodecyl sulfate–polyacrylamide gel electrophoresis (SDS-PAGE) gel, a single band with a molecular mass of ~ 130 kDa was observed in the fraction no. 21 eluted from gel filtration chromatography (Supplementary Fig. 8a,b). A single band was also observed on the native PAGE gel, and its corresponding region showed the highest enzyme activity (Supplementary Fig. 8c,d). Based on these results, *G. adiacens* secreted 130 kDa HA lyase extracellularly (Supplementary Fig. 8e).

**Table 1:** Summary of *G. adiacens* HA lyase purification.

Step	Volume (mL)	Total protein (mg)	Total activity (unit)	Specific activity (unit/mg)	Yield (%)	Purification (fold)
Supernatant	2000	57.7	26.8	0.5	100	1.0
Ammonium sulfate precipitation	125	14.9	14.7	1.0	55	2.1
DEAE-650M	6.6	1.0	8.8	8.6	33	18.6
Superdex 200 pg	0.06	0.004	0.1	24.8	0.4	53.3

**Figure 7:**
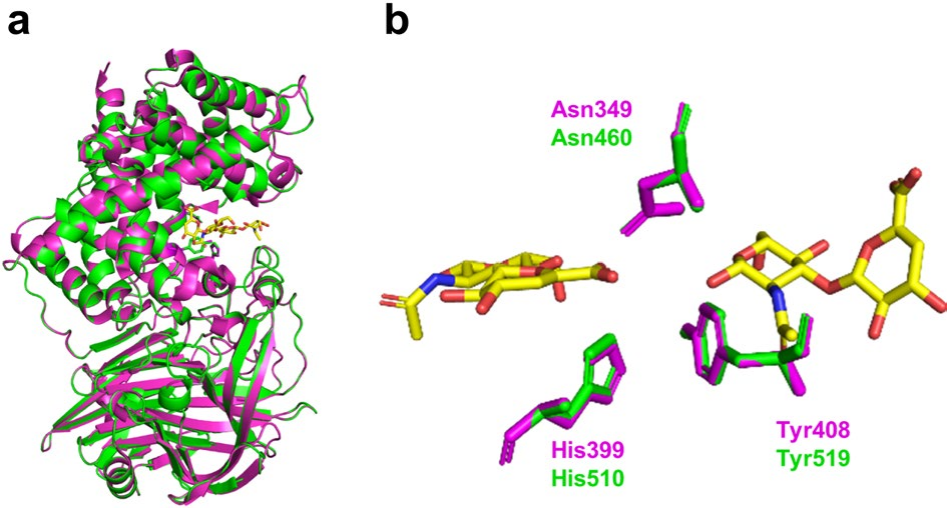
Comparison of HA lyases of *G. adiacens* and *S. pneumoniae*. (**a**) SWISS-MODEL homology modeling of LPXTG-motif cell wall anchor domain protein of *G. adiacens* (GadHL), based on the crystal structure of HA lyase of *S. pneumoniae* (SpnHL2, PDB ID 1c82). (**b**) Superimposition of the catalytic sites for HA lyase activity. Green, GadHL; magenta, SpnHL2; yellow, HA disaccharide units. Homology modeling suggested the conservation of the catalytic sites for the GadHL.

The purified HA lyase was digested with trypsin to identify the enzyme gene in the bacterial genome. A trypsin-cleaved peptide fragment, IVFLGSEVK, was mapped to the LPXTG-motif cell wall anchor domain protein of 125.6 kDa in *G. adiacens* strain ATCC 49175 (GenBank accession no. EEW37614), and this protein showed high sequence similarity to previously reported HA lyases in pathogenic *Streptococcus pneumoniae* (hereafter referred to as SpnHL1 and SpnHL2 for GenBank accession no. VOZ20400 and PDB ID 1c82, respectively; Supplementary Fig. 9). Based on the primary structure, the tertiary structure of the *G. adiacens* protein was modeled by a program of SWISS-MODEL^37^ (Fig. 7a). Previous studies revealed Asn349, His399, and Tyr408 as catalytic sites for the HA lyase activity of SpnHL2^38,39^. These amino acid residues and their steric arrangement were fully conserved in the LPXTG-motif cell wall anchor domain protein of *G. adiacens* (Fig. 7b), which was termed as GadHL. The N-terminal 32 amino acids were predicted as a signal peptide for the extracellular secretion of GadHL (Supplementary Fig. 9).

### A novel type of *Granulicatella* GAG genetic cluster

**Figure 8:**
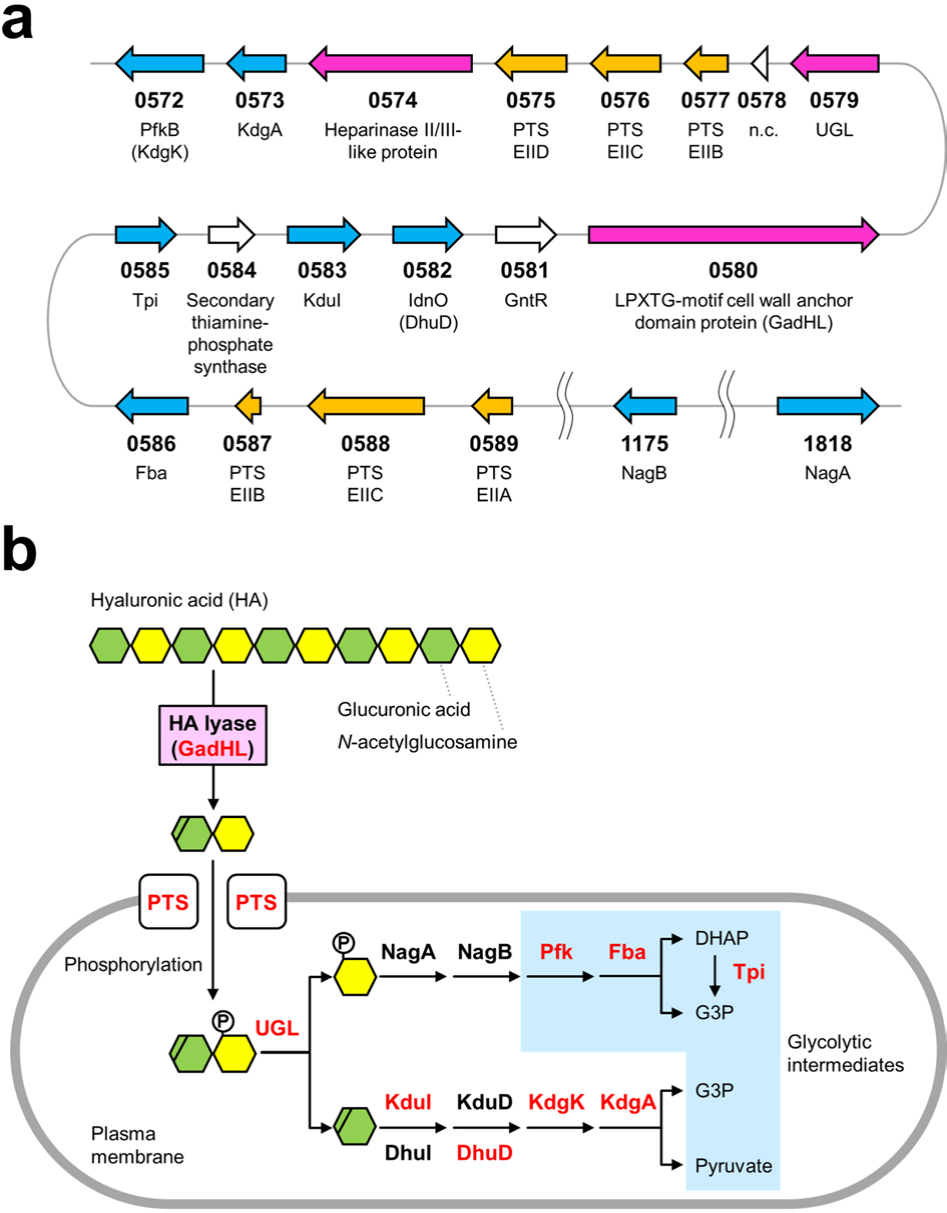
Action of GAG (HA) by *G. adiacens*. (**a**) GAG genetic cluster, including the *gadHL* and neighboring genes. Pink, genes for GAG degradation; orange, genes for substrate import by phosphotransferase system (PTS); blue, genes for GAG metabolism. (**b**) GAG assimilation model in *G. adiacens*. Red letters indicate proteins encoded by the genetic cluster found in this study. A novel type of GAG genetic cluster was found in some *Granulicatella* genomes.

The GadHL-encoding gene (*gadHL*) was found in scaffold 1 (GenBank accession no. GG694015) of the total genome of *G. adiacens* ATCC 49175. The *gadHL* and neighboring genes formed a similar GAG genetic cluster for HA import, degradation, and metabolism (Fig. 8a), i.e., genes encoding PTS, UGL, KduI, KdgA, and glycolytic enzymes [triosephosphate isomerase (Tpi), fructose-1,6-bisphosphate aldolase (Fba), and fructose-6-phosphate kinase B (PfkB)]. In the HA metabolic pathway, no genes encoding *N*-acetylglucosamine-6-phosphate (GlcNAc6P) deacetylase (NagA), glucosamine-6-phosphate (GlcN6P) deaminase (NagB), KduD/DhuD, and KdgK were annotated in the vicinity of the *gadHL* gene. Two genes encoding NagA and NagB, crucial for the metabolism of an amino sugar (i.e., GlcNAc) released from unsaturated HA disaccharides, were located in scaffold 2 (GenBank accession no. GG694016) and scaffold 4 (GenBank accession no. GG694018), respectively. The *pfkB* and *idnO* genes annotated in the vicinity of *gadHL* were suggested to encode KdgK (identity of 48.4%) and DhuD (identity of 79.6%), respectively, through primary sequence analysis. The genes involved in HA degradation and assimilation were mostly included in this genetic cluster (Fig. 8b).

The gene organization of the GAG genetic cluster in *G. adiacens* was novel in that (i) genes encoding KduI and DhuD were assembled to a single cluster and (ii) genes encoding Pfk, Fba, and Tpi involved in the metabolism of amino sugar were included in the cluster. Both KduI and DhuI exhibit 4-deoxy-l-*threo*-5-hexosulose-uronate ketol-isomerase activity, although there was little sequence identity between them. KduD and DhuD were also mutually isozyme for 2-keto-3-deoxy-d-gluconate dehydrogenase. To the best of the authors’ knowledge, only two combinations, KduI/KduD and DhuI/DhuD, were encoded in the bacterial GAG genetic cluster (Supplementary Fig. 1b). In contrast, *G. adiacens* included genes encoding for KduI and DhuD in the GAG genetic cluster (Fig. 8a).

GlcNAc6P is produced from unsaturated HA disaccharides through successive reactions by PTS and UGL (Fig. 8b). GlcNAc6P is converted to GlcN6P by GlcNAc6P deacetylase (NagA), and the resultant GlcN6P is converted to glucose-6-phosphate (Glc6P) by GlcN6P deaminase (NagB). Glc6P is metabolized to two molecules of glyceraldehyde-3-phosphate by glycolytic enzymes such as Pfk, Fba, and Tpi. Therefore, except for NagA and NagB, almost all proteins or enzymes involved in GAG (HA) degradation, import, and metabolism were encoded in a single genetic cluster (Fig. 8a,b). Because no report on the *pfk*/*fba*/*tpi*-included GAG cluster was analyzed so far, GAG genetic clusters were compared among *Granulicatella* species (Supplementary Fig. 10). Similar GAG genetic clusters were conserved in some *Granulicatella* species. Similar to *G. adiacens*, *Granulicatella* sp. strains HMSC31F03 and HMSC30F09 included genes encoding Fba and Tpi in their GAG genetic cluster. Variations in gene combinations and compositions had probably arisen through molecular evolution for a bacterial survival strategy.

## Conclusion

This is the first report on resident oral bacteria utilizing HA as a target nutrient for survival and adaptation in the oral environment. Due to GAGs in the oral environment, such as saliva and gingiva, these polysaccharides are excellent nutrients for human oral microbiota. In fact, (i) oral-abundant bacteria belonging to three of eight typical genera tested degraded GAGs, (ii) oral microbiota showed GAG-degrading and/or GAG-assimilating ability, and (iii) bacterial GAG-degrading species were easily isolated from oral microbiota. Therefore, GAGs are considered one of the host determinants for formation/composition of human oral microbiota. Among human oral microbiota, *G. adiacens* with a peculiar GAG genetic cluster was extremely propagated by assimilation of HA.

## Materials and methods

### Materials

HP, CSC, and HA were purchased from Nacalai Tesque, Fujifilm Wako Pure Chemical Co., and Sigma-Aldrich, respectively. HA with a molecular mass of 1500–1800 kDa produced from *Streptococcus equi* was used in this study. Gifu anaerobic medium (GAM) was from Nissui Pharmaceutical Co. All other analytical grade chemicals used in this study are commercially available.

### Preparation of human oral microbiota

Oral samples were kindly given as gifts by Japanese volunteers. The samples were collected by rubbing the surface of the teeth and gingiva of volunteers (males in their 20’s, 30’s, and 50’s), who had not put anything in their mouth for 6 h or more, with a sterilized swab and suspending it in saline. Saliva samples for 16S rDNA amplicon sequence analysis were also obtained from the same three volunteers. Informed consent was acquired from all subjects, and experiments using the samples were approved by the Committee of Research Activity Promotion of Graduate School of Agriculture, Kyoto University (No. H30-6). All methods were performed in accordance with relevant guidelines/regulations.

### Bacterial strains and culture conditions

As typical oral bacteria, eight following strains were purchased from the Japan Collection of Microorganisms (JCM; Tsukuba, Japan) or the NITE Biological Resource Center (NBRC; Kisarazu, Japan): *A. oris* JCM 16131, *C. pseudodiphtheriticum* JCM 1320, *N. mucosa* JCM 12992, *P. glucanolyticus* NBRC 15330, *P. dentalis* JCM 13448, *P. goodfellowii* JCM 16774, *S. oralis* subsp. *oralis* JCM 12997, and *T. denticola* JCM 8152. The bacterial strains or the oral microbiota were routinely grown in nutrient-rich liquid GAM [1% (w/v) peptone, 0.3% soy peptone, 1% proteose peptone, 1.35% digested serum powder, 0.5% yeast extract, 0.22% meat extract, 0.12% liver extract, 0.3% glucose, 0.25% potassium dihydrogen phosphate, 0.3% sodium chloride, 0.5% soluble starch, 0.03% l-cysteine hydrochloride, and 0.03% sodium thioglycolate (pH 7.1)]. *A. oris*, *P. dentalis*, *P. goodfellowii*, *S. oralis* subsp. *oralis*, and *T. denticola* were grown at 37 °C under anaerobic conditions using Anaero Pack, Anaerobic cultivation sets (Mitsubishi Gas Chemical; Tokyo, Japan). *C. pseudodiphtheriticum* and *N. mucosa* were aerobically grown at 37 °C. *P. glucanolyticus* was also aerobically grown at 30 °C. Note that the isolated *G. adiacens* strains from oral microbiota were grown in the presence of 0.001% pyridoxal hydrochloride. To investigate degradation and assimilation of GAG, *G. adiacens* was grown in nutrient-poor medium (1/20 × GAM) containing HA at 37 °C for 2 days to express HA-degrading enzymes.

### GAG degradation assay

Oral samples, cell suspensions, or concentrated culture supernatants were subjected to GAG degradation assays as follows: After centrifugation of bacterial culture, the cell pellet was washed and suspended in 200 μL sterilized saline. The supernatant was filtrated using a 0.2 μm filter and subjected to ammonium sulfate precipitation. Precipitated proteins were dissolved in 15 μL of 20 mM Tris–HCl (pH 7.5). Fifteen microliters of oral samples, cell suspensions, or concentrated culture supernatants were spotted on the center of the halo-forming minimal medium plate [0.1% yeast extract, 0.1% potassium dihydrogen phosphate, 0.1% disodium hydrogen phosphate, 0.01% magnesium sulfate heptahydrate, 0.1% ammonium sulfate, 0.04% l-cysteine hydrochloride, 1% bovine serum albumin (BSA), 0.2% dialyzed GAG (HP, CSC, or HA), and 1% agar (pH 7.0)]. After the plate was incubated for several days to allow full bacterial growth, 1 mL of 2 M acetic acid was spread on the plate to form white precipitation of the BSA-GAG complex. In GAG-degrading samples, a halo (clear zone) was observed on the plate due to the lack of the GAG polymer.

### Assimilation assay

Precultured bacterial cells were inoculated into 20-fold diluted (1/20 ×) GAM supplemented with 0.1% potassium dihydrogen phosphate, 0.1% disodium hydrogen phosphate, 0.01% magnesium sulfate heptahydrate, 0.1% ammonium sulfate, and 0.04% l-cysteine hydrochloride, with or without 0.2% glucose or dialyzed HA (pH 7.0), followed by further cultivation at 37 °C under anaerobic condition. The oral microbiota suspended in saline was inoculated into minimal medium [0.1% yeast extract, 0.1% potassium dihydrogen phosphate, 0.1% disodium hydrogen phosphate, 0.01% magnesium sulfate heptahydrate, 0.04% l-cysteine hydrochloride, and 0.1% ammonium sulfate (pH 7.0)] with or without 0.2% dialyzed GAG (CSC or HA), followed by further cultivation at 37 °C under anaerobic or aerobic condition. Bacterial cell growth was monitored by measuring OD_600_ of the culture broth. HA concentrations in the culture broth were enzymatically determined based on the calibration curve using HA as a standard. Briefly, HA was completely degraded by streptococcal HA lyase^22^, and the degraded products were monitored by measuring the absorbance at 235 nm derived from the C–C double bond in the products.

### 16S rDNA amplicon sequence analysis

Human saliva samples were inoculated into the minimal medium with or without 0.2% glucose or HA. After anaerobic or aerobic culture at 37 °C for 1 day, cells were washed with sterilized saline and immediately frozen at − 80 °C.

Both DNA extraction and 16S rDNA amplicon sequence analysis were performed by TechnoSuruga Laboratory Co. (Shizuoka, Japan; for donor #1 samples) or Bioengineering Lab. Co., Ltd. (Sagamihara, Japan; for donors #2 and #3 samples) based on a previously reported method^40^. In summary, the V3-V4 region of 16S rDNA was amplified using the 341F/R806 primer sets. Sequencing was conducted using a paired-end, 2 × 300 bp cycle run on a MiSeq sequencing system (Illumina) and MiSeq Reagent Kit version 3 (600 cycles) chemistry. After the sequencing was done, image analysis, base calling, and error estimation were done using Illumina Real-time Analysis (version 1.17.28). Paired-end sequencing with read lengths of ~ 430 bp was done as well. Succeeding the demultiplexing, a clear overlap in the paired-end reads was seen. This made paired reads be joined together with the fastq-join program. Only reads with quality value scores of ≥ 20 for > 99% of the sequence were extracted for supplemental analysis. Metagenome@KIN software (World Fusion) was utilized for homology searching with the determined 16S rDNA sequences. Bacterial species were then identified based on the data from 97% similarity cutoff with DB-BA13.0.

### Protein and enzyme assays

Concentrations of proteins or enzymes were determined by the Bradford method^41^ using BSA as a standard protein. HA lyase was incubated in a reaction mixture containing 0.05% HA as a substrate and 50 mM potassium phosphate (pH 7.5). The enzyme activity was determined by monitoring the increase in absorbance at 235 nm. Absorbance at 235 nm was derived from the C–C double bond of the reaction products, i.e., unsaturated oligosaccharides released from HA. The molar absorption coefficients of unsaturated HA oligosaccharides at 235 nm [ε_235_ (M^−1^ cm^−1^)] were estimated to be 5500. One unit of enzyme activity was defined as the amount of enzyme required to release 1 µmol of the product/min.

### Electrophoresis

Protein purity was confirmed by SDS-PAGE^42^, followed by Coomassie brilliant blue (CBB) or silver staining. Native PAGE^43^ was also carried out to identify a protein band with the enzyme activity.

### Purification and identification of HA lyase from *G. adiacens*

The cells of *G. adiacens* were aerobically cultured to the stationary phase in 1/20 × GAM containing 0.05% HA at 37 °C for 2 days. To investigate the localization of HA lyase, extracellular and intracellular fractions were prepared as follows. The culture broth was centrifuged at 9700×*g* and 4 °C for 5 min. The resultant supernatant was used as an extracellular fraction. After washing with saline and recentrifugation, the precipitants were suspended in saline, and the cell suspension was used as an intracellular fraction.

For enzyme purification, 2 L culture supernatant was treated with ammonium sulfate to 90% saturation. The mixture was stirred overnight and centrifuged at 9300×*g* for 30 min. The precipitate was dialyzed against 20 mM Tris–HCl (pH 7.5). The dialysate was loaded onto a TOYOPEARL DEAE-650M column (Tosoh) equilibrated with 20 mM Tris–HCl (pH 7.5). After washing unabsorbed proteins with the same buffer, the column was eluted with a linear gradient of 0–1 M sodium chloride in 20 mM Tris–HCl (pH 7.5). The active fractions were concentrated with Centriprep-10K (Merck Millipore). The concentrate was applied onto a HiLoad 16/60 Superdex 200 pg column (GE Healthcare) equilibrated with 20 mM Tris–HCl (pH 7.5) containing 150 mM sodium chloride. The active fractions were concentrated with Centriprep-10K and Vivaspin 500 (Sartorius).

Trypsin-digested peptides derived from purified HA lyase were analyzed by Japan Proteomics Co., Ltd. (Sendai, Japan) using nanoflow liquid chromatography-tandem mass spectrometry.

## Supplementary Information

Supplementary Figures.

## Data Availability

All sequences determined in the 16S rDNA amplicon sequence analysis have been deposited in the DNA Data Bank of Japan (DDBJ) under the accession number DRA011872. The nucleotide sequences of 16S rDNA from HA-degrading bacteria isolated from teeth and gingiva have been deposited in the DDBJ under accession numbers LC646145 and LC646146, respectively.
