# Supplementary material for: Enhanced propagation of Granulicatella adiacens from human oral microbiota by hyaluronan
Source: Sci Rep. 2022 Jun 29;12:10948. doi: 10.1038/s41598-022-14857-9 (PMC9243090; doi:10.1038/s41598-022-14857-9)
Supplement: Supplementary file 1 — Supplementary Figures. [file 41598_2022_14857_MOESM1_ESM.pdf]

Supplementary Information

Enhanced propagation of *Granulicatella adiacens* from human oral microbiota by hyaluronan

Shun Yabuuchi, Sayoko Oiki, Shuma Minami, Ryuichi Takase, Daisuke Watanabe, and Wataru Hashimoto

Laboratory of Basic and Applied Molecular Biotechnology, Division of Food Science and Biotechnology, Graduate School of Agriculture, Kyoto University, Uji, Kyoto 611-0011, Japan

Correspondence and request for materials should be addressed to W.H. (e-mail: hashimoto.wataru.8c@kyoto-u.ac.jp).

Supplementary Fig. 1 Degradation of GAG by bacteria.

Supplementary Fig. 2 GAG degradation by model oral bacteria on nutrient-rich medium.

Supplementary Fig. 3 TLC assays for GAG degradation assays by human oral microbiota.

Supplementary Fig. 4 CSC assimilation by human oral microbiota.

Supplementary Fig. 5 Growth curves for metagenomic analysis.

Supplementary Fig. 6 Metagenomic analysis of human oral microbiota cultivated under different carbon sources.

Supplementary Fig. 7 HA degradation and assimilation by *G. adiacens*.

Supplementary Fig. 8 Purification of *G. adiacens* HA lyase.

Supplementary Fig. 9 Alignment of amino acid sequences of HA lyases identified from *G. adiacens* (GadHL) and *S. pneumoniae* (SpnHL1 and SpnHL2).

Supplementary Fig. 10 The HA-utilizing gene cluster highly conserved among *Granulicatella* species.

Supplementary Fig. 1

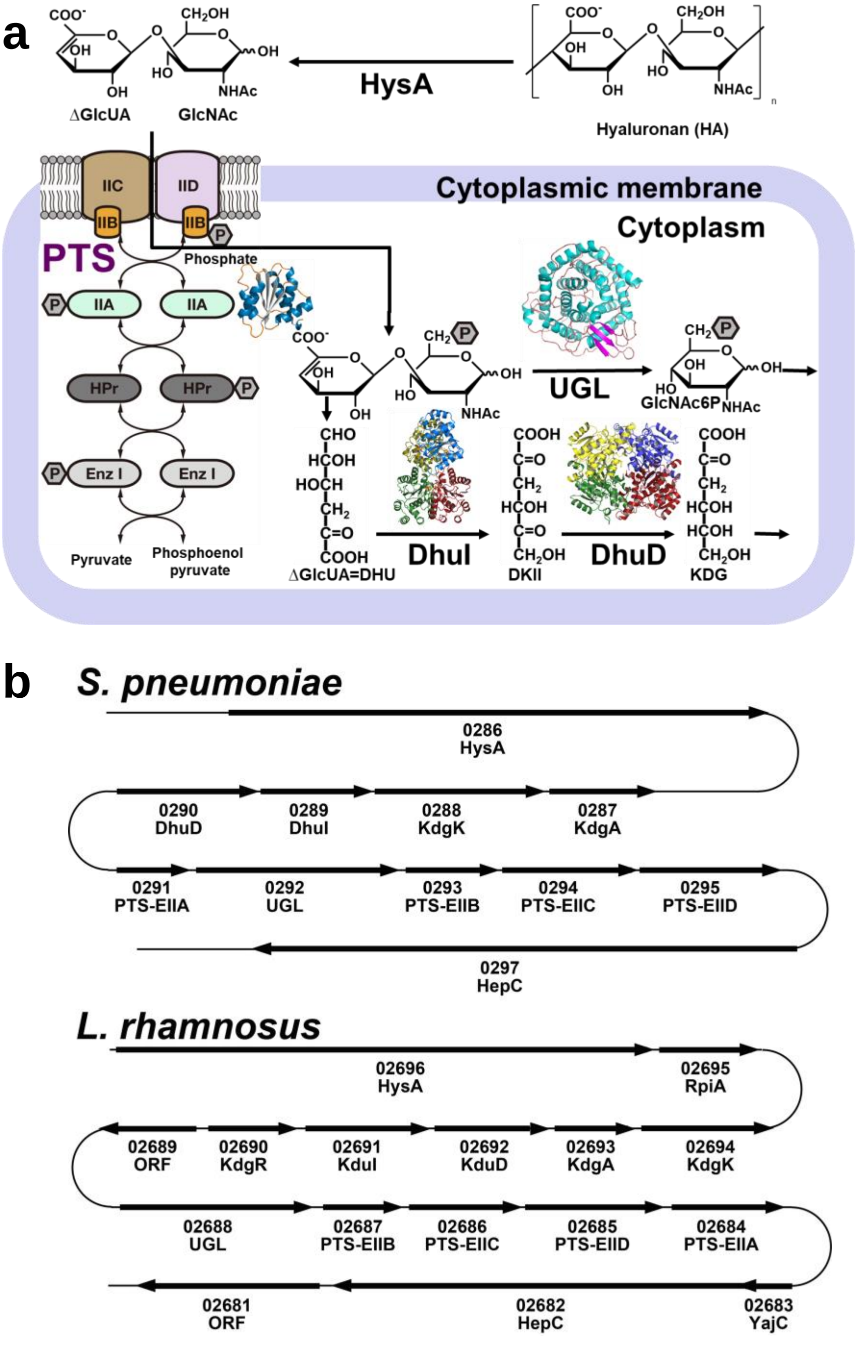

Supplementary Fig. 1 Degradation of GAG by bacteria. a GAG degradation/import/metabolism of GAG (HA) by streptococci. HA is depolymerized to unsaturated disaccharides by extracellular/cell-surface HA lyase (HysA). The resultant HA disaccharides are incorporated to cytoplasm by PTS through phosphorylation, and degraded to constituent monosaccharides (i.e. unsaturated GlcUA and GlcNAc) by hydrolase (UGL). Unsaturated GlcUA is nonenzymatically converted to Dhu, which is further metabolized by isomerase (DhuI) and reductase (DhuD). **b** GAG genetic cluster. Upper, *S. pneumoniae* strain R6; lower, *Lactobacillus rhamnosus* strain Lc705. Four or five digits indicate gene ID numbers of each bacterium (spr#### in *S. pneumoniae* and Lc705_##### in *L. rhamnosus*). Gene products (see text) are shown under gene ID. This figure is cited from ref. 25 after slight modification.

Supplementary Fig. 2

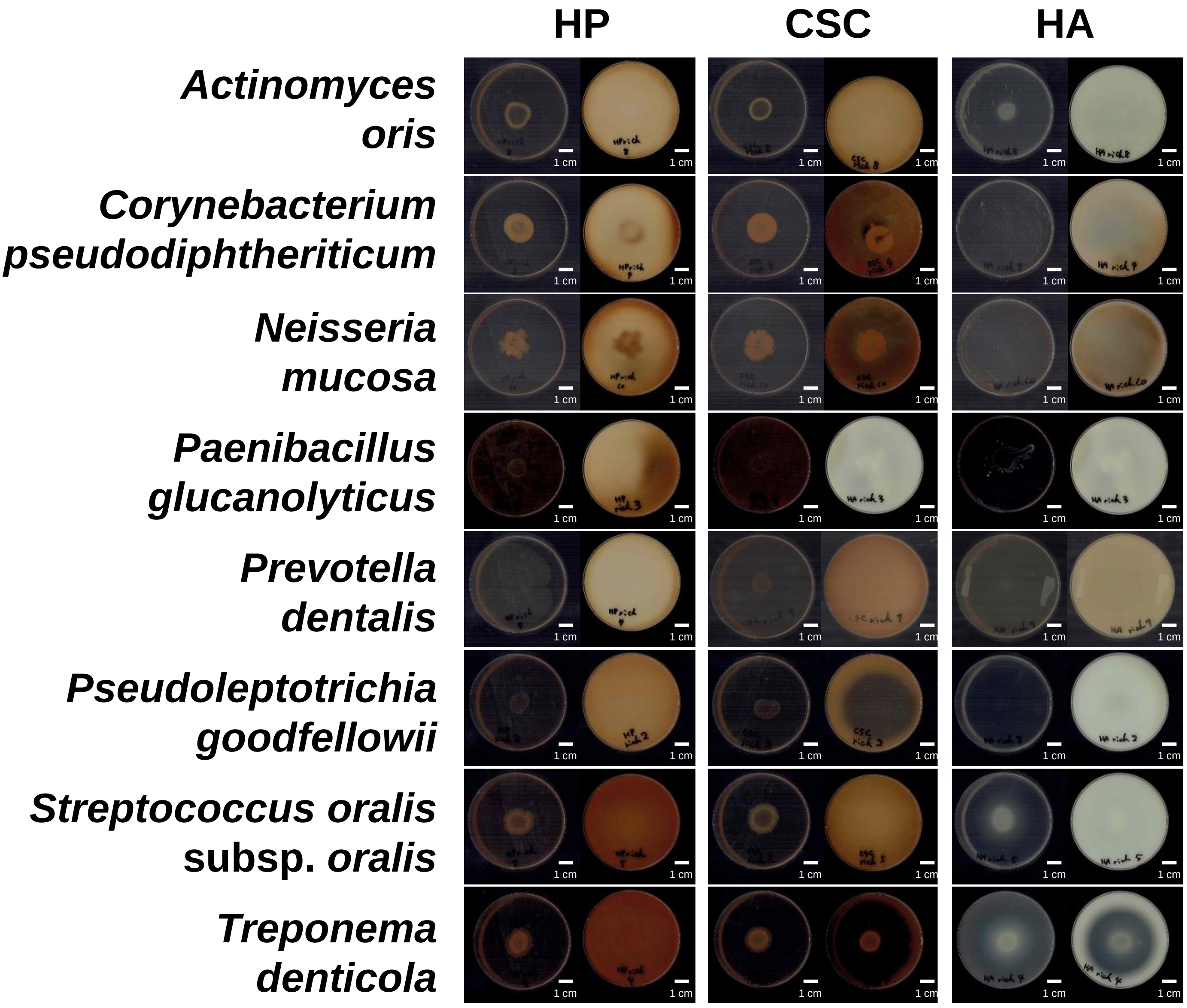

Supplementary Fig. 2 GAG degradation by model oral bacteria on nutrient-rich medium. Eight model oral bacterial species from different genera were grown on the center of the halo-forming nutrient-rich medium plate containing HP (left panels), CSC (middle panels), or HA (right panels). The HP- or CSC-containing medium is GAM with 0.2% HP or CSC, 1% BSA, and 1.5% agar. The HA-containing medium is consisted of 0.1% yeast extract, 0.1% potassium dihydrogen phosphate, 0.1% disodium hydrogen phosphate, 0.01% magnesium sulfate heptahydrate, 0.1% ammonium sulfate, 5% glucose, 0.04% L-cysteine hydrochloride, 1% BSA, 0.2% HA, and 1.5% agar After full growth on the medium plate (left), acetic acid was spread onto the plate for halo formation (right). Bars, 1 cm.

Supplementary Fig. 3

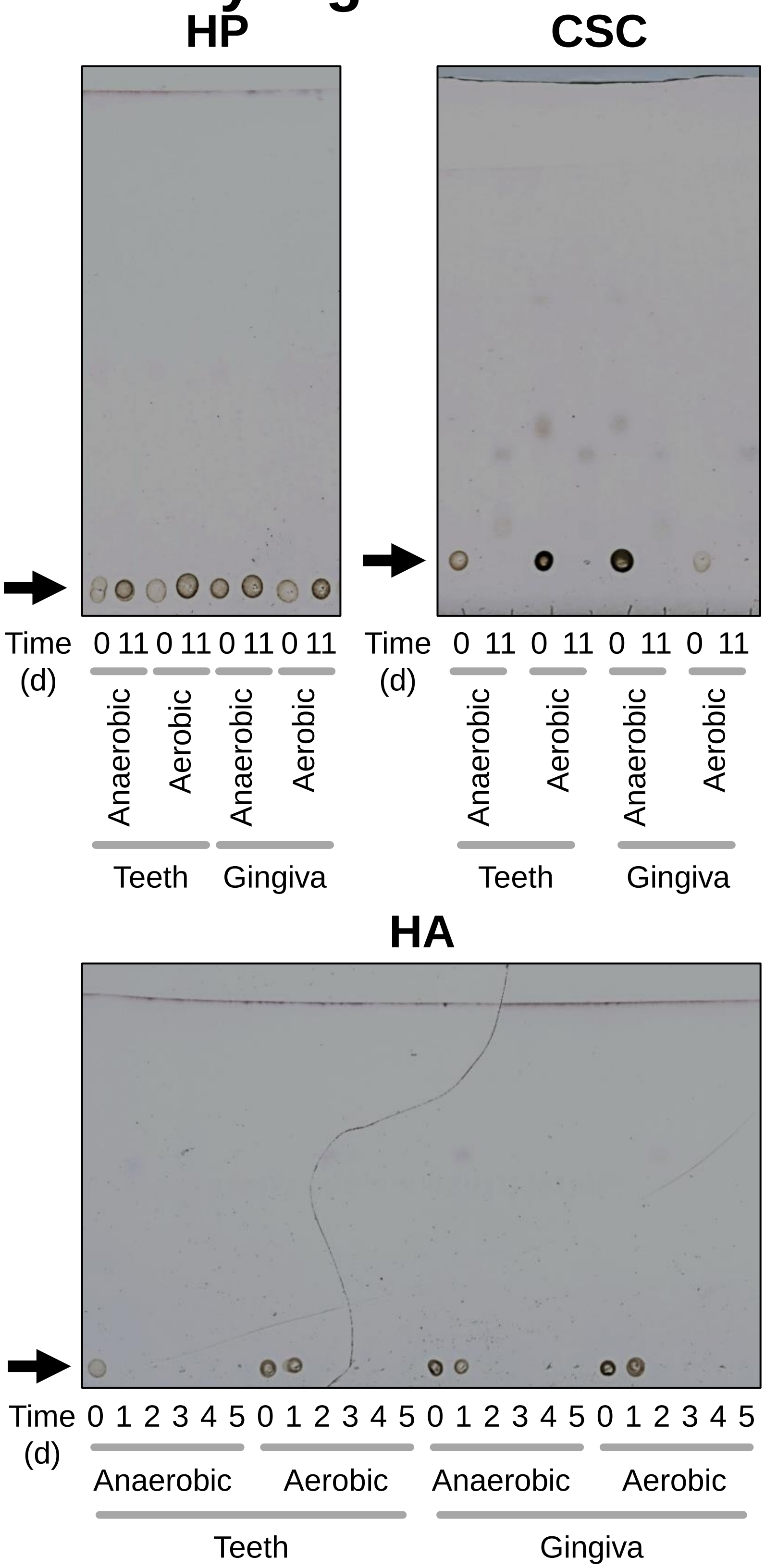

Supplementary Fig. 3 TLC assays for GAG degradation by human oral microbiota. Microbial community from the teeth or gingiva of donor #1 was grown anaerobically or aerobically in minimal liquid medium containing HP, CSC, or HA. TLC was performed using a solvent system of water:acetic acid:1-butanol (2:2:3, v:v:v). GAGs and their breakdown products were visualized by heating the TLC plates [silica gel 60 F_{254} (Merck)] at 130˚C for 5 min after spraying with ethanol containing 10% sulfuric acid. Arrows indicate the starting positions where the culture supernatant samples were spotted. No migration was observed in GAG polysaccharides due to their high-molecular-weights.

Supplementary Fig. 4

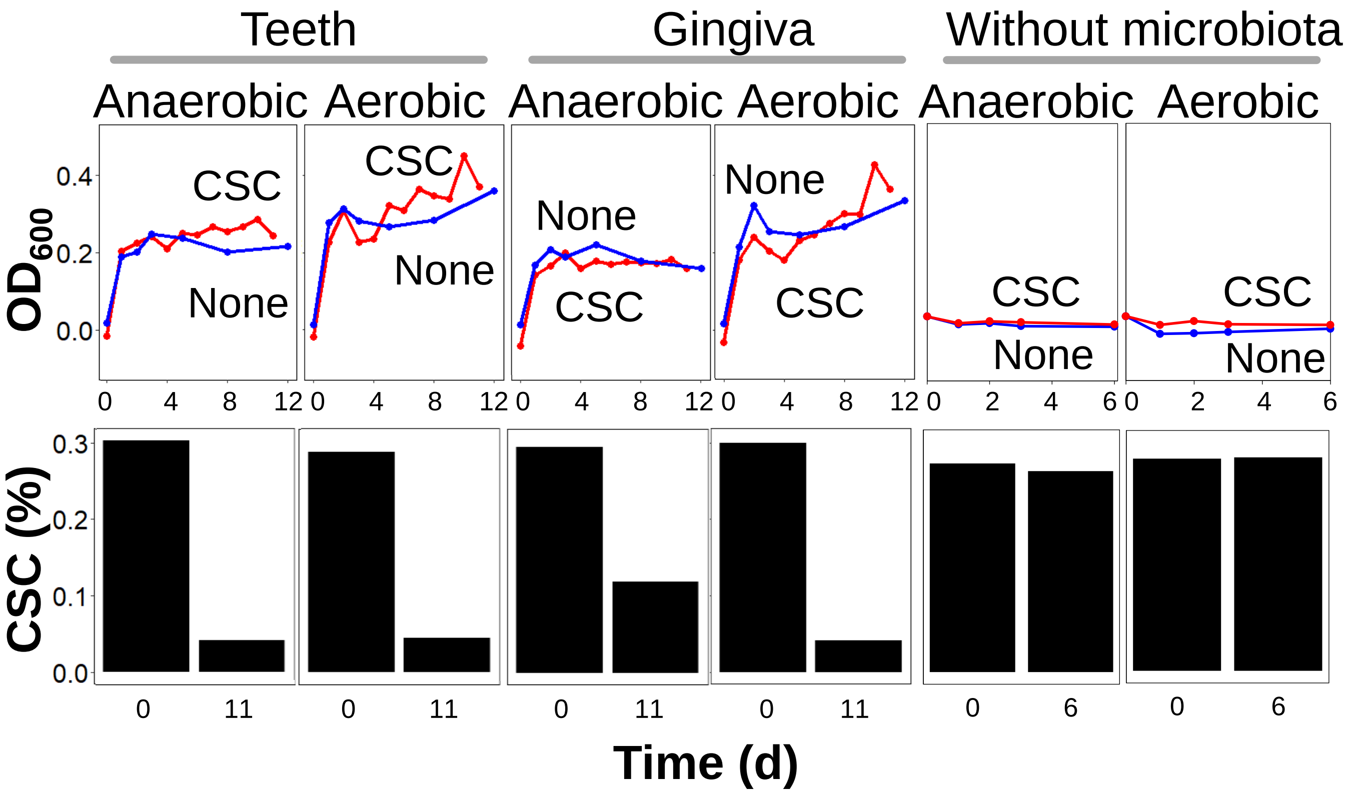

Supplementary Fig. 4 CSC assimilation by human oral microbiota. Growth of microbial community from the teeth (left) or gingiva (center) of donor #1 in low-nutrient medium (blue) or in the same liquid medium but containing CSC (red) as a carbon source was compared (upper). CSC concentrations before and after the growth test in the CSC-containing medium were determined (lower). As a negative control (right), growth and CSC concentration in the absence of microbiota were also measured.

Supplementary Fig. 5

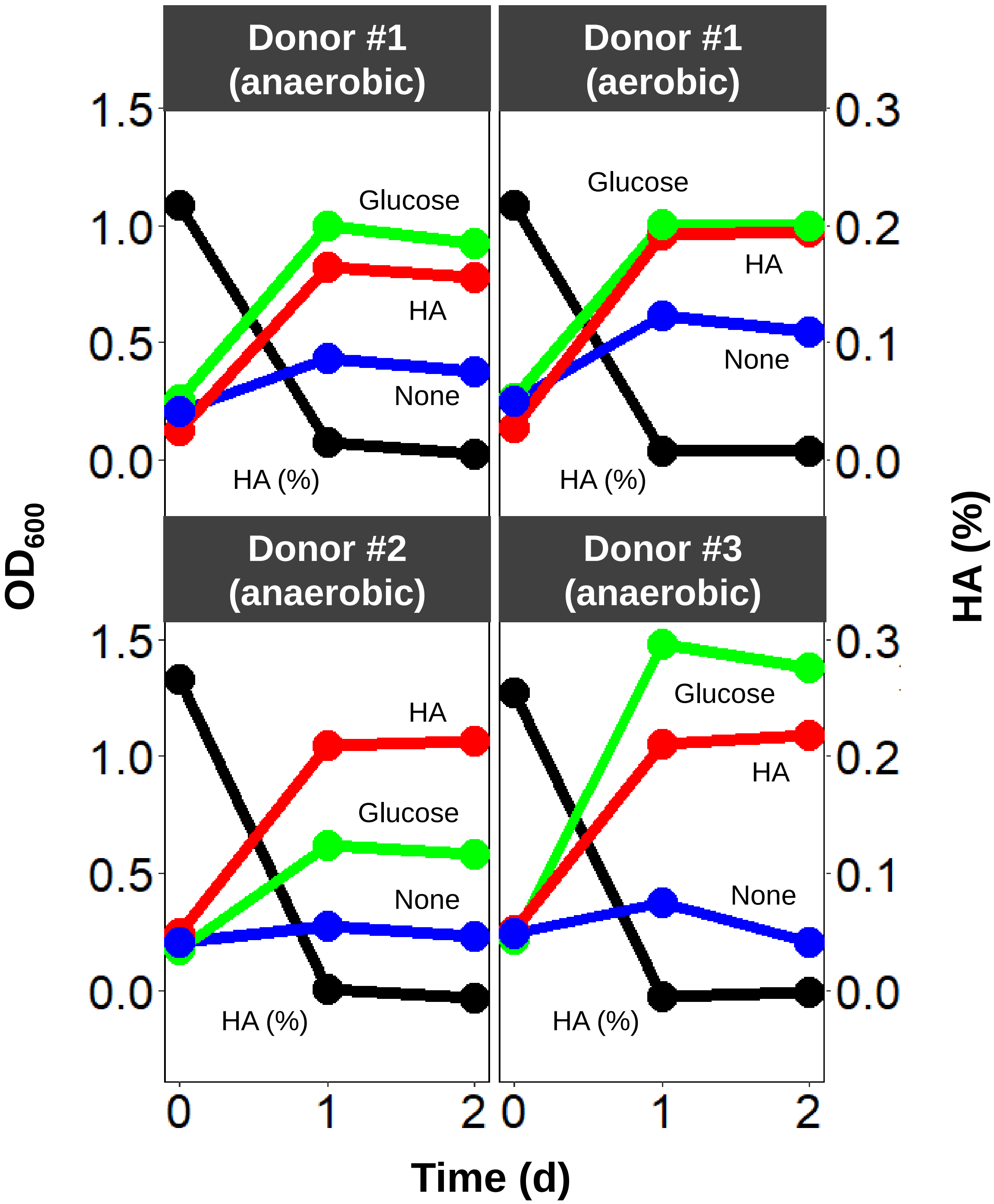

Supplementary Fig. 5 Growth curves for metagenomic analysis. Saliva samples from donors #1, #2, and #3 were anaerobically or aerobically (only for donor #1) grown in low-nutrient medium (blue) or the same liquid medium but containing glucose (green) or HA (red) as a carbon source. HA concentrations during the growth test in the HA-containing medium were determined (black).

Supplementary Fig. 6

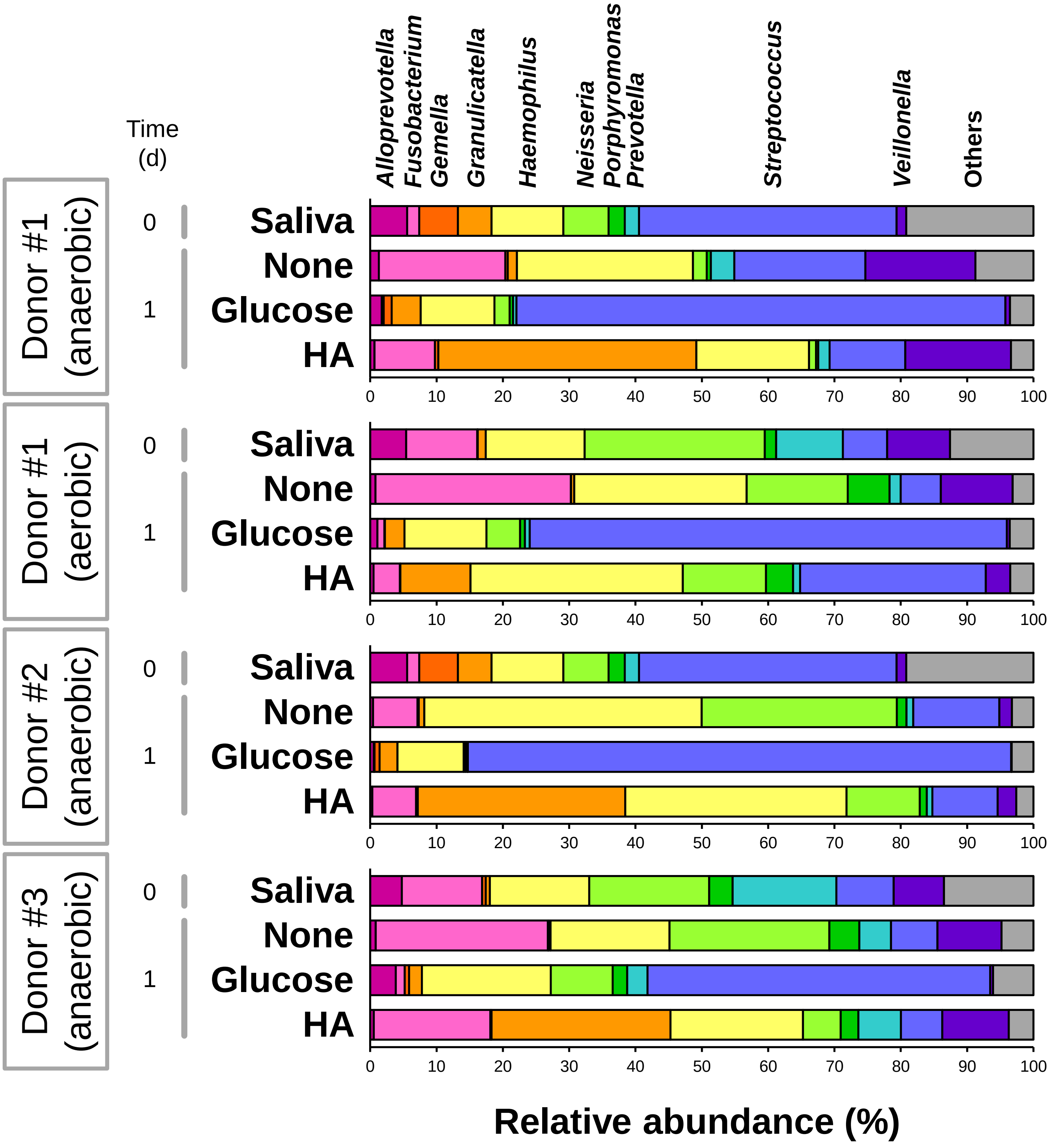

Supplementary Fig. 6 Metagenomic analysis of human oral microbiota cultivated under different carbon sources. Saliva samples from donors #1, #2, and #3 were anaerobically or aerobically (only for donor #1) grown in low-nutrient medium or the same liquid medium but containing glucose or HA as a carbon source. The saliva samples before inoculation and the microbial communities after 1-d cultivation were subjected to 16S rRNA gene-based metagenomic analysis.

Supplementary Fig. 7

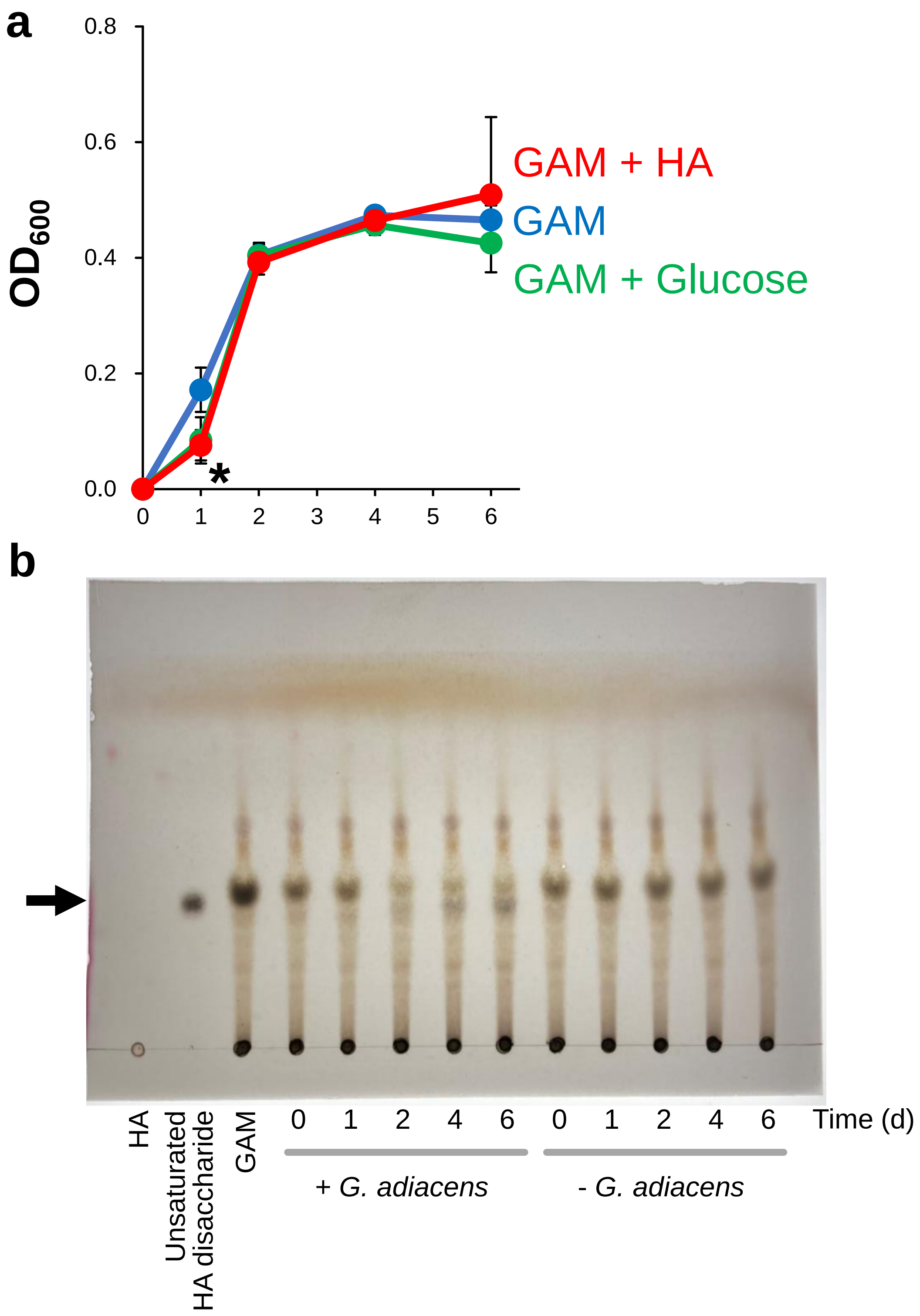

Supplementary Fig. 7 HA degradation and assimilation by *G. adiacens*. a Growth of *G. adiacens* in nutrient-rich medium (GAM; blue) or the same liquid medium but containing glucose (green) or HA (red) was compared. $*p < 0.05$, significant growth promotion compared to growth in nutrient-rich medium (GAM; t test). **b** HA and its breakdown products were visualized by TLC analysis. An arrow indicates the position of unsaturated HA disaccharides.

Supplementary Fig. 8

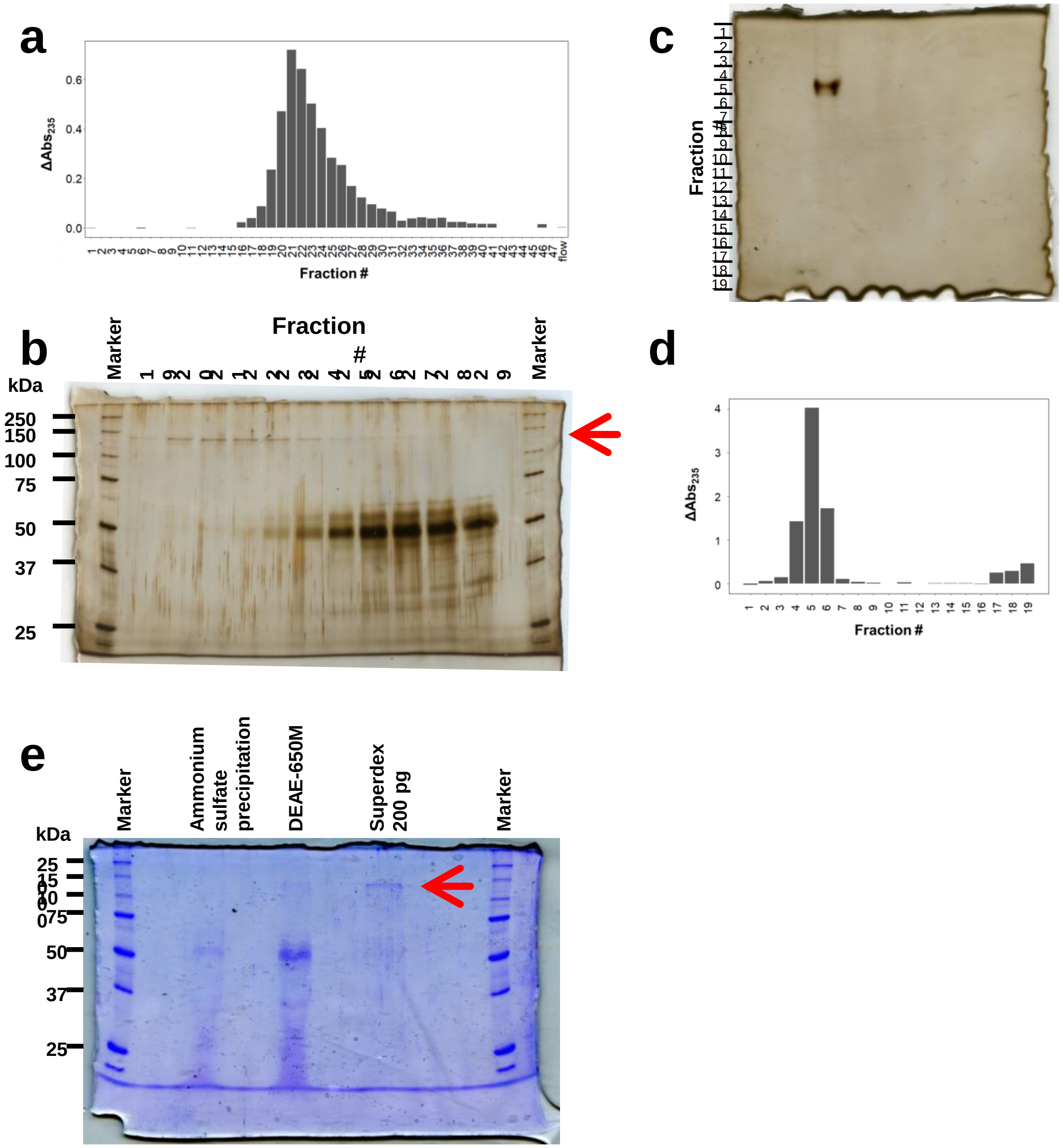

Supplementary Fig. 8 Purification of *G. adiacens* HA lyase. a Relative HA lyase activity of the eluate fractions separated by gel filtration chromatography. Lyase activity was assayed by measuring the increase in absorption at 235 nm upon cleavage of HA. **b** Proteins in the eluate fractions #19 – 29 analyzed by SDS-PAGE. After separation, proteins were visualized by silver staining. A red arrow indicates the position of an approximately 130-kDa protein band, which exhibited a good correlation with the HA lyase activity. Fractions #19 – 21 were collected for further analysis. **c** Purified HA lyase analyzed by native PAGE. After separation, proteins were visualized by silver staining. A single band was observed at fraction #5. **d** Relative HA lyase activity of the protein eluted from the individual gel fragments. Lyase activity was assayed by measuring the increase in absorption at 235 nm upon cleavage of HA. **e** Summary of *G. adiacens* HA lyase purification. Fractions after ammonium sulfate precipitation (approximately 15 μg of proteins), after anion exchange chromatography (approximately 1.5 μg of proteins), and after gel filtration chromatography (approximately 0.67 μg of proteins) were analyzed by SDS-PAGE. After separation, proteins were visualized by CBB staining. A red arrow indicates the position of an approximately 130-kDa protein band, which was subjected to trypsin digestion and nanoLC-MS/MS analysis. The original unprocessed gel images (**b**, **c**, and **e**) are shown.

Supplementary Fig. 9

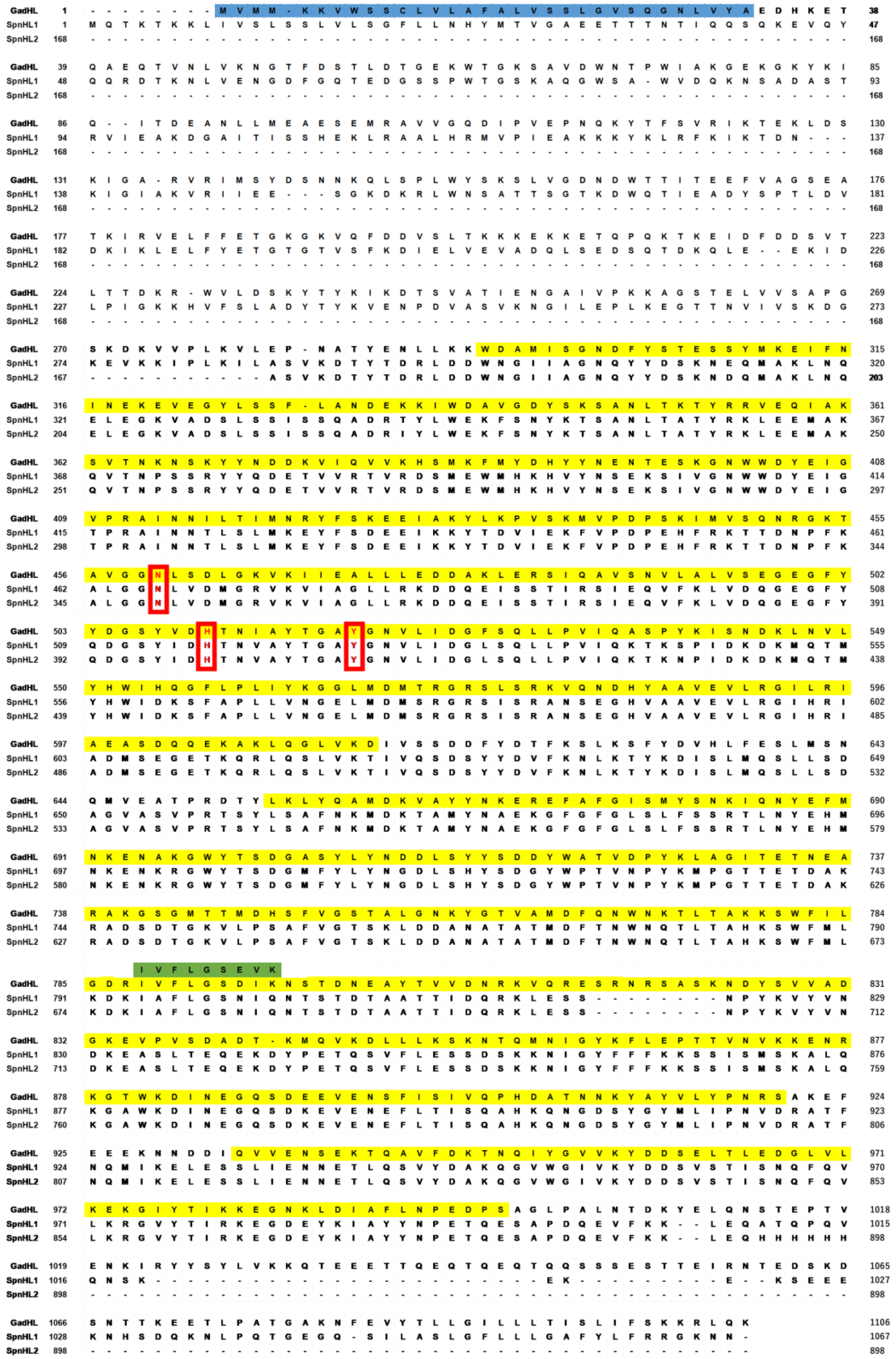

Supplementary Fig. 9 Alignment of amino acid sequences of HA lyases identified from *G. adiacens* (GadHL) and *S. pneumoniae* (SpnHL1 and SpnHL2). Yellow, HA lyase domain in GadHL; blue, predicted signal peptide in GadHL; red boxes, catalytic residue sites suggested in SpnHL2; green, the peptide fragment (IVFLGSEVK) mapped to GadHL, which was included in HA lyase purified in the present study.

Supplementary Fig. 10

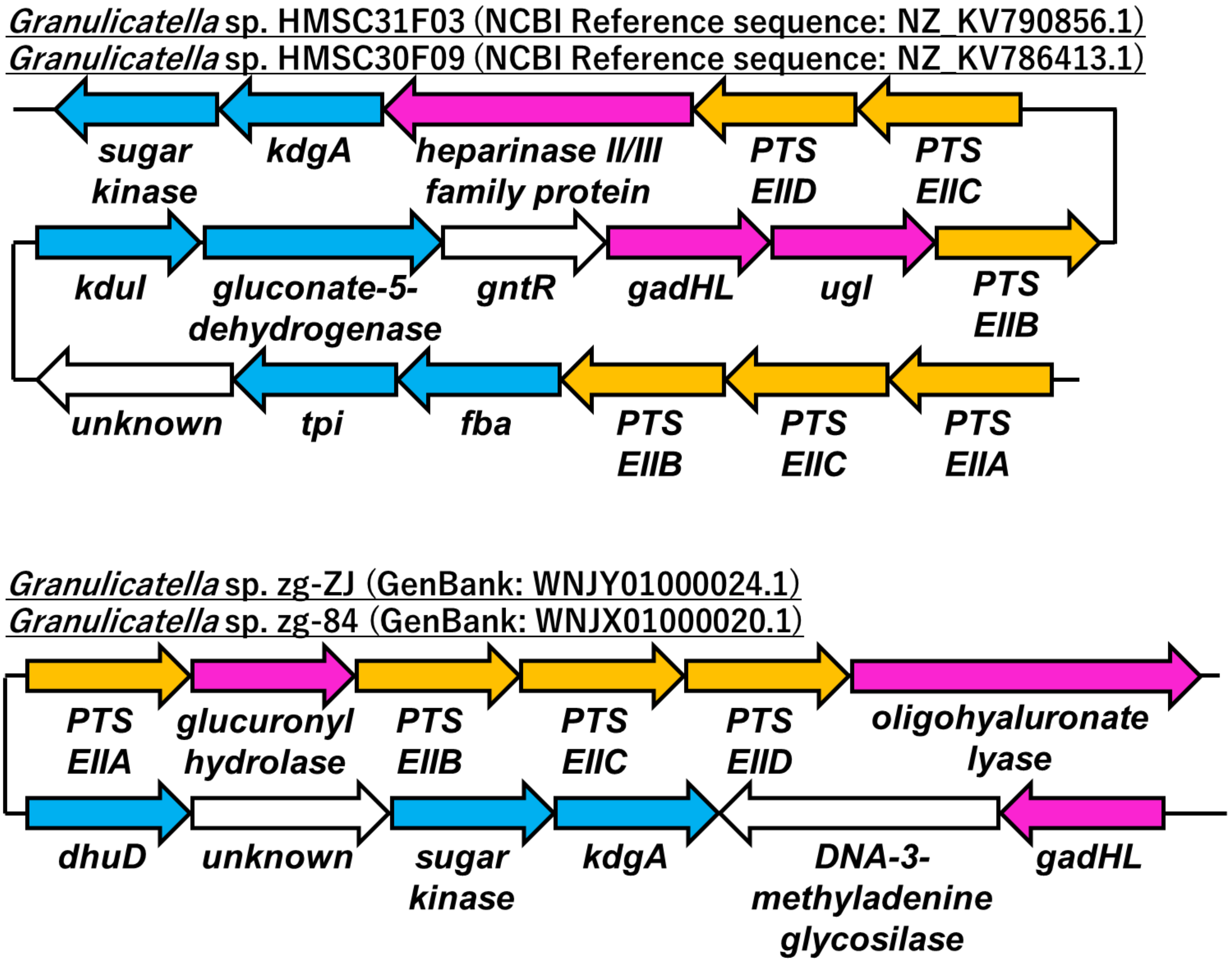

Supplementary Fig. 10 The HA-utilizing gene cluster highly conserved among *Granulicatella* species. Pink, degrading enzyme-encoding genes; orange, PTS genes for substrate transport; blue, carbon metabolic enzyme-encoding genes.
